# Usefulness of preoperative simulation with patient-specific hollow vascular models for high-flow renal arteriovenous fistula embolization using a preloading coil-in-plug technique^☆^

**DOI:** 10.1016/j.radcr.2022.07.028

**Published:** 2022-07-29

**Authors:** Ryo Morita, Daisuke Abo, Takeshi Soyama, Tetsuaki Imai, Bunya Takahashi, Yuki Yoshino, Naoya Kinota, Hiroyuki Hamaguchi, Takuto Kameda, Kohsuke Kudo

**Affiliations:** a Department of Diagnostic and Interventional Radiology, Hokkaido University Hospital, N-14, W-5, Kita-ku, Sapporo, Hokkaido, 060-8648, Japan; b Department of Diagnostic and Interventional Radiology, Kushiro City General Hospital, 1-12 Shunkodai, Kushiro, Hokkaido, 085-0822, Japan; c Department of Neurosurgery, Hakodate Central General Hospital, 33-2 Honcho, Hakodate, Hokkaido, 040-8585, Japan; d Department of Radiology, Hakodate Municipal Hospital, 1-10-1 Minatocho, Hakodate, Hokkaido, 041-8680, Japan; e Department of Radiological Technology, Hokkaido University Hospital, N-14, W-5, Kita-ku, Sapporo, Hokkaido, 060-8648, Japan; f Department of Diagnostic Imaging, Faculty of Medicine, Hokkaido University, N-15, W-7, Kita-ku, Sapporo, Hokkaido, 060-8638, Japan; g Global Center for Biomedical Science and Engineering, Faculty of Medicine, Hokkaido University, N-14, W-5, Kita-ku, Sapporo, Hokkaido, 060-8648, Japan

**Keywords:** Preoperative simulation, Patient-specific hollow vascular model, Renal arteriovenous fistula, Preloading coil in plug technique, Three-dimensional printer

## Abstract

The development of three-dimensional printers has facilitated the creation of patient-specific hollow vessel models. Preoperative simulations using these types of models have improved our ability to select appropriate devices and embolic materials before performing complex endovascular procedures. This report describes 2 cases of high-flow renal arteriovenous fistulas (r-AVFs) that were successfully treated via short-segment embolization using the preloading coil-in-plug (p-CIP) technique. To our knowledge, this is the first report of r-AVF being treated using the p-CIP technique. Our findings demonstrate that preoperative simulation has the potential to improve the safety and reliability of complex vascular embolization procedures.

## Introduction

Three-dimensional (3D) printing is a rapidly developing technology with applications relevant to human anatomical models [1]. The creation of patient-specific hollow vascular models is quicker and easier than ever before [2]. Recently, it was reported that hollow vascular models were manufactured using desktop 3D printers [3,4].

Endovascular procedure simulation using patient-specific, hollow vascular models is useful for preoperative simulation and interventional radiology training [2,5,6].

The usefulness of preoperative simulation for peripheral embolization of visceral aneurysms has been reported [7]. Because the model focuses on aneurysms to be embolized and does not include the abdominal aorta as access route, the simulation was primarily performed to select the embolization materials. However, selecting the optimal access device for the target artery, such as a catheter or a guidewire, is also necessary for a successful procedure. There has been no study of whether access devices are truly appropriate in the preoperative simulation of peripheral embolization.

Renal arteriovenous fistulas (r-AVF) in endovascular treatment are generally treatable with coil embolization [8]. To preserve renal function, the short segment of the renal artery near the fistula needs to be embolized, while sparing a normal branch of the renal artery is also necessary. However, in cases of high-flow fistulas, embolizing with coils is technically challenging due to the increased risk of migration of embolic materials [9]. In such cases, using a plug was reported to reduce the risk of migration [10]. Although plugs have facilitated short-segment embolization, long-term recanalization has been reported [11]. In order to prevent recanalization, the coil-in-plug embolization method has been developed to enhance the effectiveness of embolization [12]. In addition, recently, the use of a preloading coil-in-plug (p-CIP) technique for internal iliac artery embolization during endovascular aortic repair (EVAR) was reported as a method of reliably placing the coil in the plug [13].

Therefore, we considered embolizing in the short segment for high-flow r-AVF using the p-CIP technique to preserve as much normal renal parenchyma as possible. However, a drawback of plugs is that a large-diameter catheter tip must be advanced beyond the target lesion for plug placement, which may be difficult to introduce through tortuous access routes [8]. In addition, the risk of vascular injury increases when attempting to insert a large catheter.

Preoperative simulation using a patient-specific 3D model that includes the target embolization vessel and the access route (the iliac artery and the abdominal aorta) facilitates the selection of optimal devices, including catheters and embolization materials. Therefore, we decided to create and simulate such a vascular model that would allow us to evaluate preoperatively whether the plug could be delivered, and whether the access device would fit.

This report describes 2 cases of successful high-flow r-AVF embolization using the p-CIP technique after preoperative simulation with patient-specific hollow vascular models created by a 3D printer.

## Case presentation

### Preoperative simulation with patient-specific hollow vascular models

Our institutional review board approved the use of vascular models for this retrospective case report. Before 3D printing, Digital Imaging and Communications in Medicine (DICOM) of 3D computed tomographic angiography (CTA) images with a slice thickness of 0.5 mm were converted to stereolithography (STL) files using InVesalius 3.1.1 software (Renato Archer Information Technology Centre, São Paulo, Brazil).

The hollow feature of the model was created using a mesh mixer (Autodesk, San Rafael, CA). Wall thickness was set at 1.3 mm or 1.5 mm. Data corruption was then assessed with support provided by PreForm (Formlabs, Somerville, MA). A hollow vessel model was prepared using a Form 2 or 3 L 3D printer (Formlabs) with flexible and non-transparent/transparent materials.

**Fig. 1:**
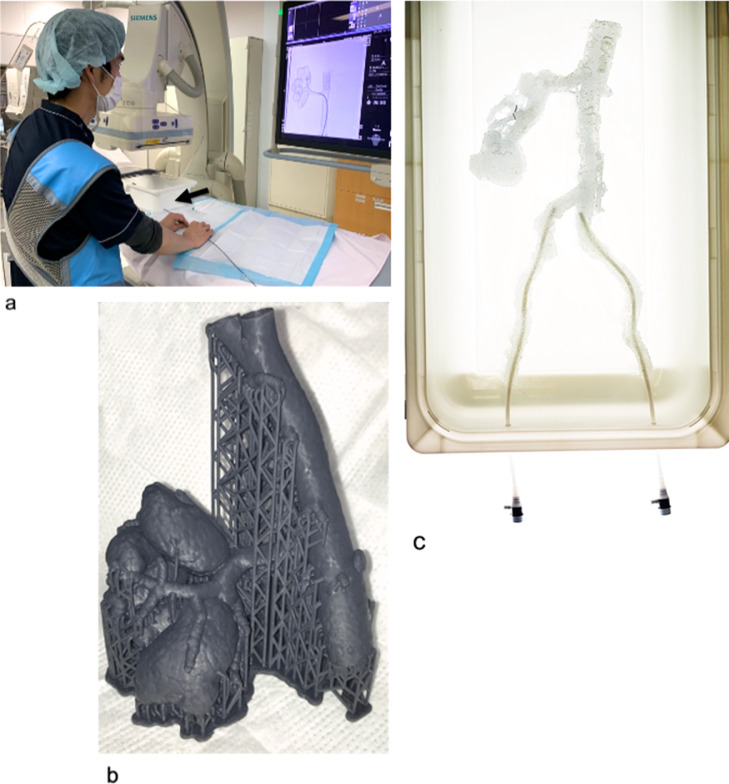
The setting of preoperative simulation with patient-specific hollow vascular models. (A) Preoperative simulation using vascular models made of non-transparent material with fluoroscopy in Case 1. The model was connected to the sheaths in a white box (black arrow). **(B)**The renal arteriovenous fistula (r-AVF) models were made of non-transparent material in Case 1. **(C)** The r-AVF models were made of transparent material in Case 2. This model was connected to sheaths in a white box.

The 3D vascular model used for Case 1 was made of flexible and non-transparent material. The model was used for preoperative simulation with fluoroscopy (Figs. 1A and B). In contrast, preoperative simulation in Case 2 was performed without fluoroscopy, using a 3D vascular model made of flexible and transparent material (Fig. 1C). Using a sprayer, the inner lumen of the vascular model in Case 2 was coated with silicone. Each model was connected to a sheath in a white box.

### Preloading coil-in-plug technique

In preparation for the procedure, a microcatheter was inserted into the Amplatzer Vascular Plug 1 (AVP1; Abbott Vascular, Redwood City, CA) outside the body, and the plug and microcatheter were inserted into the target vessels. The plug was placed at the optimal position, and then the microcatheter was pulled back into the plug. Embolization was then performed using coils. Finally, the plug was detached.

### Case 1

**Fig. 2:**
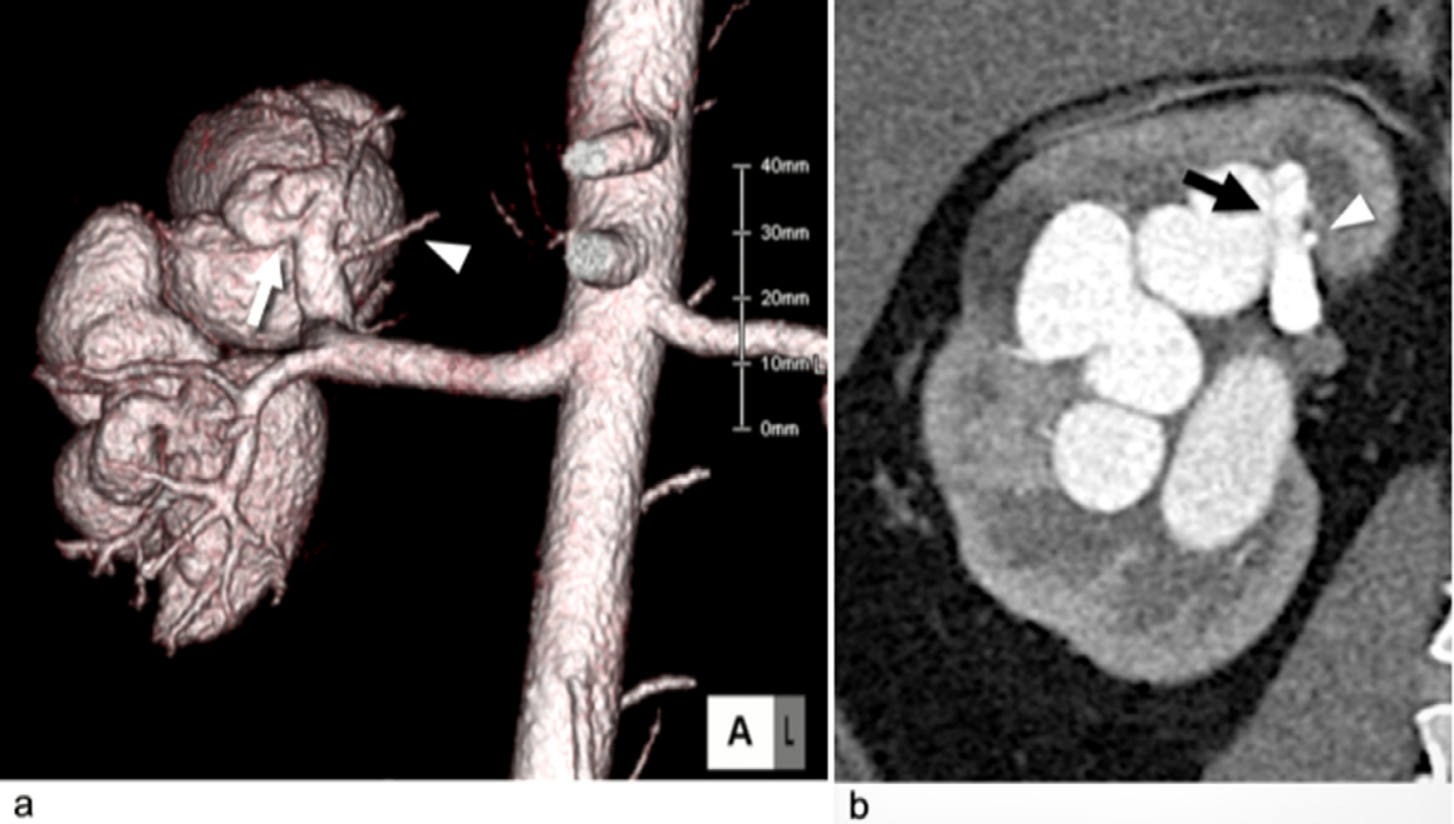
A 25-year-old male patient with right renal arteriovenous fistulas (r-AVF) in Case 1. (A) Volume rendering of computed tomography (CT) angiography showing a huge right r-AVF with a dilated venous sac near the fistula (white arrow) and early venous return. The white arrowhead indicates a normal branch proximal to the fistula. (B) An arterial-phase coronal CT image showing a huge right r-AVF with a venous sac connecting the dilated renal vein. The fistula (black arrow) is proximal to the normal branch (white arrowhead).

A 25-year-old male patient with asymptomatic high-flow right r-AVF that was found incidentally during a physical examination was admitted to our hospital for treatment to prevent heart failure. No history of trauma or renal biopsy was found. Preoperative 3D CTA revealed a large fistula at the lateral wall of the superior interlobular artery that was connected to a dilated draining vein (Fig. 2A). Embolization of the straight and short segment of the superior interlobular artery was necessary to spare the normal branch proximal to the fistula (Fig. 2B). Therefore, we attempted to embolize the segment using the p-CIP technique.

In this case, we aimed to determine the feasibility of delivering the guiding catheter to the target renal interlobular artery near the fistula. Therefore, a non-transparent 3D-printed model was fabricated for simulation using fluoroscopy (Fig. 1B).

**Fig. 3:**
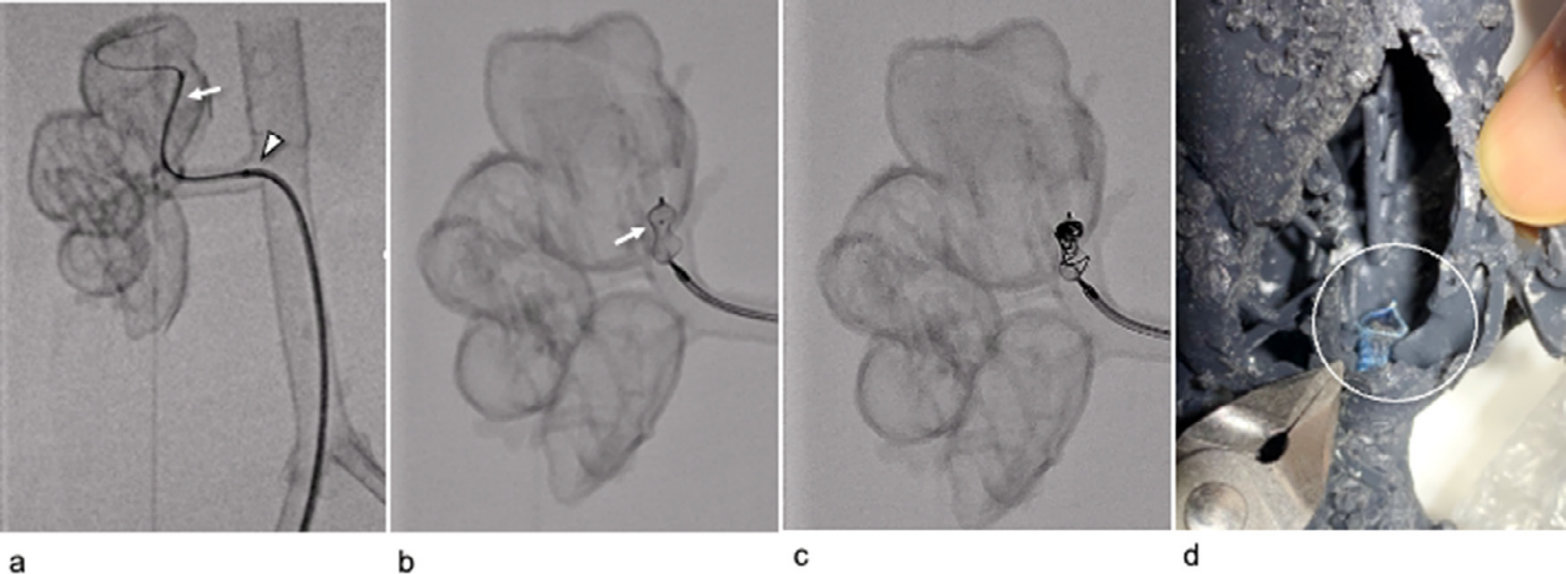
Preoperative simulation using non-transparent vascular models with fluoroscopy in Case 1. (A) A 6-Fr guiding catheter (white arrow) is advanced to the interlobar artery near the fistula using a 6-Fr guiding sheath (white arrowhead). (B) The 10-mm AVP1 and a 2.0-Fr microcatheter were advanced into the guiding catheter using the preloading coil in the plug technique. The white arrow indicates the tip of the microcatheter in the plug. (C) The lumen of the plug was embolized with a coil. (D) After embolization, the vessel model was cut to confirm that the plug (white circle) was placed in the vicinity of the fistula in the interlobar artery.

In the fluoroscopic simulation (Figs. 3A-C), a 6-Fr guiding catheter (Hyperion JR4.0 100 cm; ASAHI, Aichi, Japan) was advanced to the interlobar artery near the fistula via a 6-Fr guiding sheath (Parent hook-type 53 cm; Medikit Co., Tokyo, Japan). Subsequently, a 10-mm AVP1 and 2.0-Fr microcatheter were advanced into the guiding catheter, followed by embolization using the p-CIP technique. The vessel model was cut after embolization and plug placement in the vicinity of the interlobar artery fistula was confirmed (Fig. 3D). We tried another 6-Fr guiding catheter (Cerulean DD6 straight 108 cm; Medikit Co.), but it was too soft, making it difficult to deliver the plug.

**Fig. 4:**
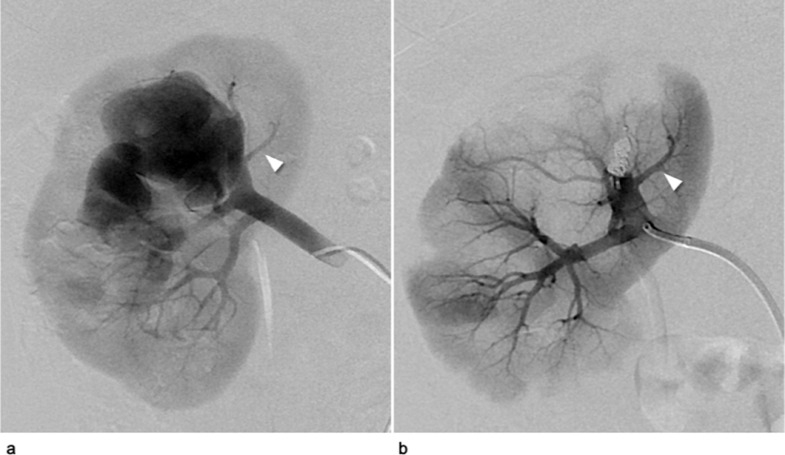
Digital subtraction angiography (DSA) during the actual treatment of Case 1. (A) DSA prior to the patient's treatment demonstrated a huge right renal arteriovenous fistula with a venous sac and a dilated vein via the fistula in the upper interlobular renal artery. The white arrowhead indicates a normal branch proximal to the fistula. (B) DSA after treatment demonstrates the complete disappearance of the fistula, and the sparing of the normal branch (white arrowhead).

Following the simulation, we decided to treat the patient using the p-CIP technique. The 10-mm AVP1 was successfully placed at the optimal position within the target interlobular artery using the same devices that were used in the simulation. The lumen of the plug was embolized with coils (AZUR18 4 mm/20 cm × 2; Terumo, Tokyo, Japan). A comparison between final angiography immediately after embolization and preoperative angiography revealed the complete disappearance of the fistula, with the normal branch proximal to the fistula being spared (Figs. 4A and B). No major complications were noted, and no recanalization was observed 18 months after treatment.

**Fig. 5:**
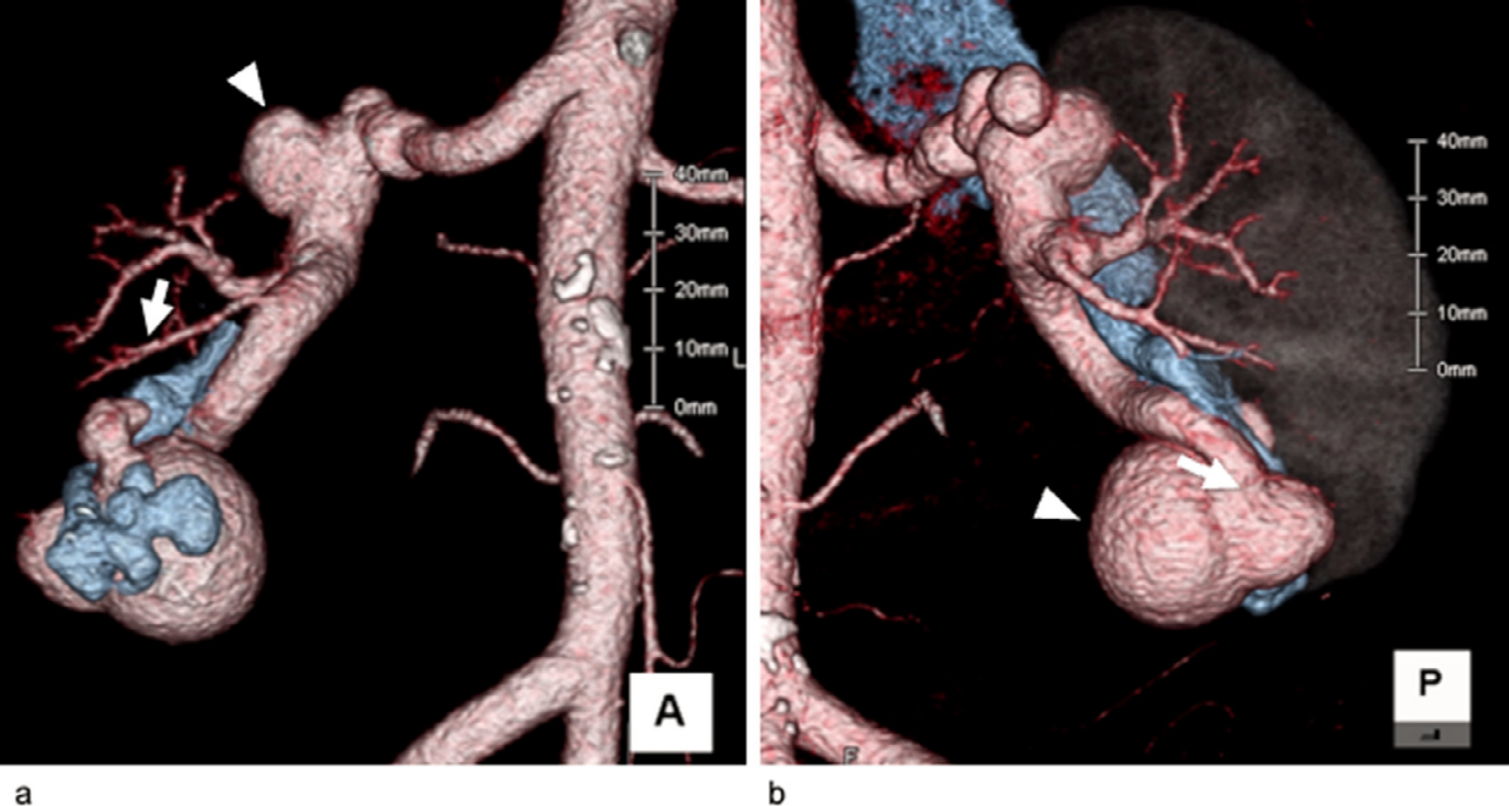
A 68-year-old female patient with right renal arteriovenous fistulas (r-AVF) in Case 2. (A) Volume rendering of computed tomography (CT) angiography showing a huge right r-AVF with small flow-related aneurysms (white arrowhead). The inferior interlobular feeding artery is dilated, and the white arrow indicates a normal branch proximal to the fistula. (B) Volume rendering of CT angiography (posterior view) showing a huge right r-AVF with early venous return. The large fistula (white arrow) at the dilated inferior interlobular artery terminal was connected to the venous sac (white arrowhead).

### Case 2

A 68-year-old woman presented with a high-flow right r-AVF and small renal aneurysms; she was followed for 11 years by her previous hospital, but an enlargement of the r-AVF was noted on CT. She also had hematuria and hypertension. Preoperative 3D CTA revealed a large fistula at the terminal end of the inferior interlobar artery that was connected to a venous sac and a dilated draining vein (Figs. 5A and B). Using the same treatment strategy as was used in Case 1, we attempted to embolize a straight, short segment of the inferior interlobar artery to spare the normal branch proximal to the fistula via the p-CIP technique. Small aneurysms were not treated, since rupture risk would likely decrease after embolization of the fistula.

A transparent 3D-printed model was fabricated for simulation without fluoroscopy (Fig. 1C). The target vessel to be embolized was larger than that in Case 1; therefore, insertion of a larger guiding system was needed. Various devices and embolization materials were tried, including catheters, sheaths, guidewires, and plugs. The following combination of devices and materials was selected.

**Fig. 6:**
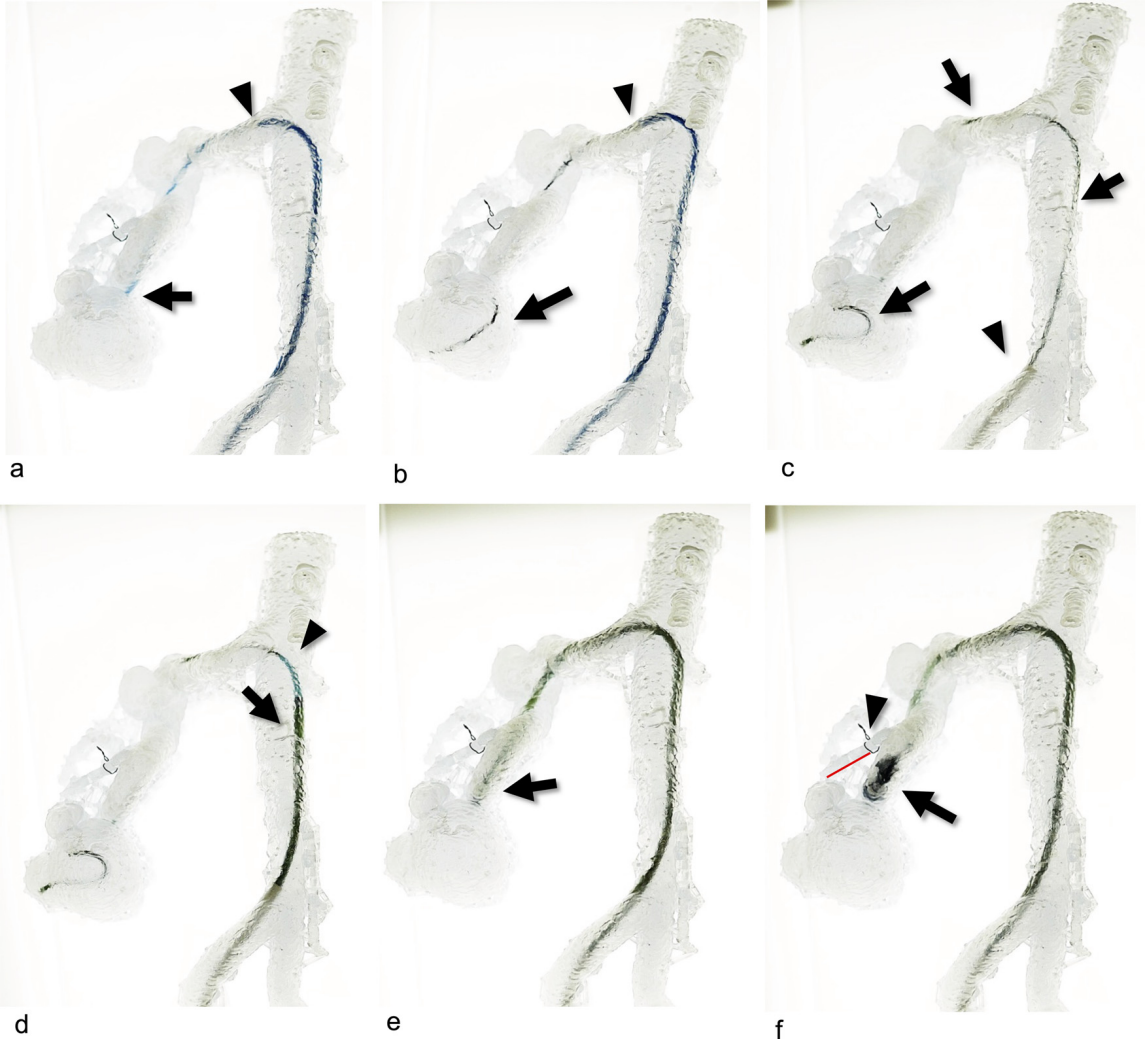
Preoperative simulation using transparent vascular models without fluoroscopy in Case 2. (A) The 4-Fr Cobra catheter (black arrow) and hydrophilic guidewire using a 5-Fr J sheath (black arrowhead) advancing to the dilated inferior interlobular artery near the fistula via the 9-Fr sheath. (B) The reshaped 0.035-inch spring wire (black arrow) was inserted via wire exchange, and the 4-Fr Cobra catheter was removed. The black arrowhead shows a 5-Fr J sheath. (C) The 5-Fr J sheath was removed, and a 0.035-inch spring wire (black arrow) via a 9-Fr sheath (black arrowhead) is shown. (D) The 6-Fr guiding sheath (black arrow) with a dilator (black arrowhead) is advanced using the wire technique. (E) The tip of the 6-Fr guiding sheath (black arrow) is placed at the interlobar artery near the fistula. (F) The 16-mm AVP1 (black arrow) was advanced to a location beyond the normal branch (red line) proximal to the fistula. A black wire (black arrowhead) wrapped around the normal branch was used as a landmark.

During simulation without fluoroscopy (Fig. 6), a 5-Fr J sheath (55 cm: Medikit Co.) and 4-Fr shepherd hook catheter (80 cm: Medikit Co.) were advanced to the right main renal artery via a 9-Fr sheath (30 cm: Medikit Co.). Second, a 4-Fr Cobra catheter (80 cm: Medikit Co.) and 0.035-inch hydrophilic guidewire via a 5-Fr J sheath were advanced to the inferior interlobar artery near the fistula (Fig. 6A). We exchanged the hydrophilic guidewire with a reshaped 0.035-inch moderate stiff spring wire (Amplatze extra-stiff wire 145 cm; Cook Medical, Bloomington, IN) via a 4-Fr Cobra catheter (Figs. 6B and C). Then, a 6-Fr guiding sheath (Destination 90 cm; Terumo) with a dilator was advanced into the target segment of the artery (Figs. 6D and E). Subsequently, a 16-mm AVP1 and a 2.0-Fr microcatheter were inserted to the optimal position via a 6-Fr guiding sheath using the p-CIP technique (Fig. 6F). We tried 2 type 4 plugs (AVP1, 12 mm/16 mm;AVP2, 12 mm/16 mm) after inserting a 6-Fr guiding sheath. AVP1 plugs could be delivered, but AVP2 could not, because the right renal artery was tortuous.

**Fig. 7:**
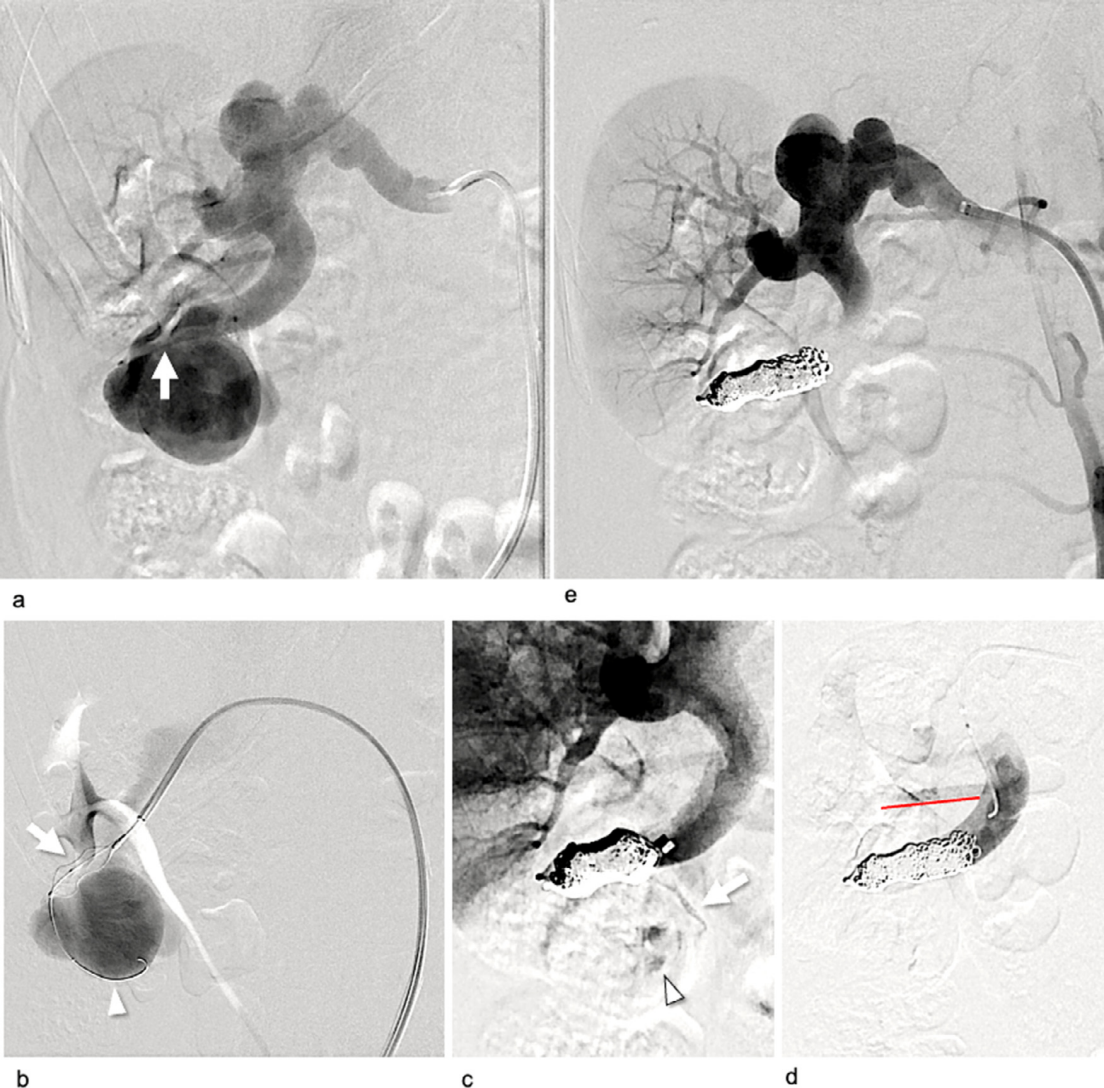
Digital subtraction angiography (DSA) during the actual treatment of Case 2. (A) DSA before treatment demonstrates a large right renal arteriovenous fistula with early venous return. Small aneurysms of the main renal artery and dilated inferior interlobular artery were connected to the large fistula (white arrow) and a venous sac. (B) The 16-mm AVP1 (white arrow) was advanced to a location near the fistula via the 6-Fr guiding sheath. The white arrowhead shows the microcatheter and microwire penetrated using the preloading coil-in-plug (p-CIP) technique. (C) After plug placement using the p-CIP technique, the residual blood flow in the venous sac (white arrowhead) via a minor feeder (white arrow) became apparent. (D) DSA via the microcatheter after additional coil embolization proximal to the plug showing the disappearance of the residual blood flow, and the sparing of the normal branch proximal to the fistula (white arrowhead). (E) DSA of the main renal artery after treatment demonstrated complete disappearance of the fistula.

**Fig. 8:**
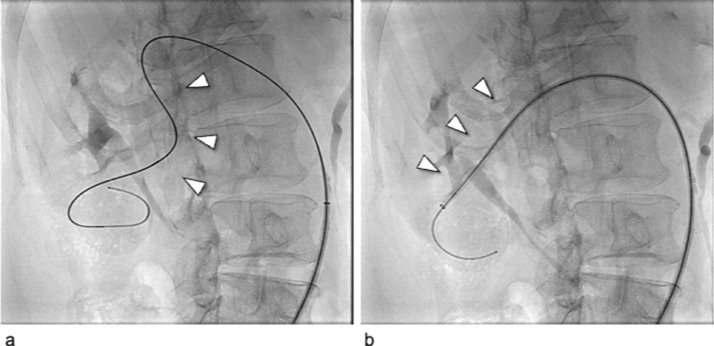
The fluoroscopic images of the deformation of the right renal artery during the actual treatment of Case 2. (A) The reshaped 0.035-inch spring wire (white arrowhead) was inserted following the tortuous renal artery, and the 6-Fr guiding sheath was advanced using the wire technique. (B) The tortuous renal artery straightened after the 6-Fr guiding sheath was inserted into the inferior interlobular artery near the fistula.

After the simulation, the patient was treated via a p-CIP technique. The 16-mm AVP1 was placed at the optimal position within the inferior interlobular artery near the fistula using the same equipment as used in the simulation (Figs. 7A-C). However, unlike during the simulation, a vessel deformity was caused by guiding sheath insertion (Fig. 8). The plug lumen was embolized with coils (AZUR18 4 mm/20 cm × 5, Target XL soft 4 mm × 12 cm × 2, 3 mm × 9 cm × 2). After plug placement, a minor feeder that was not visible on the preoperative CT became apparent (Fig. 7C). Additional pushable coils were embolized proximal to the plug (Fig. 7D). Final angiography revealed the complete disappearance of the fistula with a spared normal final branch (Fig. 7E). No major complications were noted. Further, the patient's hematuria disappeared, and hypertension improved. No recanalization of the fistula was observed 4 months post-treatment.

## Discussion

Two cases of high-flow r-AVF were successfully embolized using the p-CIP technique after preoperative simulation. Few cases of r-AVF treatment using plugs have been reported [10,11]. To our knowledge, this is the first report of r-AVF embolization using the p-CIP technique. In Case 1, for short embolization using the p-CIP technique, a large-diameter catheter needed to be guided to its optimal location beyond the normal branch. In Case 2, the right renal artery extended downward and was tortuous; therefore, it was unclear whether a large-diameter catheter would reach its optimal location.

Thus, preoperative simulation was performed after creating 3D models of the renal arteries of each patient using 3D printing. Using the models, various devices and embolization materials were tested to facilitate the selection of the optimal devices and materials for each procedure. Embolization using the p-CIP technique was accomplished using the same devices and embolization materials used in simulations. In addition, there was no need to change devices during patient treatment.

The preoperative simulation group in EVAR reported a shorter procedure time and decreased fluoroscopy time and volume of contrast medium compared to the control group [14]. We believe the benefits of simulation to patient treatment outcomes are the same in our report. Furthermore, the risk of complications, including vascular injury related to large diameter catheter insertion, was minimized by using the exact same devices and materials for patient treatment preoperatively.

The usefulness of EVAR preoperative simulation has been reported regarding stent-graft placement, including access devices such as sheaths, catheters, and guidewires [14]. To accurately simulate endovascular treatment in the abdominal area via a femoral approach, it is necessary to reproduce the iliac artery and the abdominal aorta, which affect the acceptability of access devices. Although creating a long length of the aorta-only model is possible with desktop 3D printers [1,14], it was technically difficult to create a continuous vessel model from the aorta included in the approach route to the target lesions such as peripheral visceral aneurysms [15].

Previous reports on preoperative simulation of abdominal visceral aneurysms have reproduced the aneurysm and the inflow and outflow vessels without the abdominal aorta and the iliac artery, which mostly influence the appropriate choice of access device [7]. To select the appropriate access device, it is necessary to accurately reproduce large vascular models that include the target embolization vessel and the device insertion pathway (femoral/brachial/radial approach). We were able to fabricate such a vascular model, which allowed us to perform accurate preoperative simulations and thus successfully treat the patients. In addition, the model used in Case 2 has the advantage of being transparent, allowing simulation without fluoroscopy [3].

A limitation of this study is that characteristics of the 3D vascular model including blood flow, vessel wall elasticity, and luminal surface slippage will differ from those of biological blood vessels. In fact, in Case 2, deformation of the vessel was observed (Fig. 8), an occurrence which was not predicted by the model simulation. Further studies investigating the differences between models and biological vessels are needed.

Preoperative simulation with patient-specific hollow vascular models created by a 3D printer allows physicians to perform complex vascular embolization procedures safely and reliably. Further, this methodology has potential applications that extend to other areas of endovascular treatment.

## Statement of human and animal rights

This article does not contain any studies with human or animal subjects.

## Patient consent

Informed consent: Written informed consent was obtained from the patients for publication of this case report and any accompanying image. Consent for publication: Consent for publication was obtained for every individual person's data included in the study.
